# Supplementary material for: FATE-MAP predicts teratogenicity and human gastrulation failure modes by integrating deep learning and mechanistic modeling
Source: Nat Commun. 2026 Feb 19;17:3327. doi: 10.1038/s41467-026-69596-6 (PMC13065977; doi:10.1038/s41467-026-69596-6)
Supplement: Supplementary file 1 — Supplementary Information [file 41467_2026_69596_MOESM1_ESM.pdf]

Supplementary Information

FATE-MAP predicts teratogenicity and human gastrulation failure modes by integrating deep learning and mechanistic modeling

Joseph Rufo,[1,2,3] Chongxu Qiu,[1] Dasol Han,[1,3] Naomi Baxter,[1] Gabrielle Daley,[1] Jasmine Dhillon,[1] Felix Wong[4], James J. Collins[5,6,7], and Maxwell Z. Wilson[1,2,3,4*]

[1] Department of Molecular, Cellular, and Developmental Biology, University of California Santa Barbara, Santa Barbara, CA, USA
[2] Center for BioEngineering, University of California Santa Barbara, Santa Barbara, CA, USA
[3] Neuroscience Research Institute, University of California Santa Barbara, Santa Barbara, CA, USA
[4] Integrated Biosciences, Inc., Redwood City, CA, USA
[5] Infectious Disease and Microbiome Program, Broad Institute of MIT and Harvard, Cambridge, MA, USA
[6] Institute for Medical Engineering and Science and Department of Biological Engineering, Massachusetts Institute of Technology, Cambridge, MA, USA
[7] Wyss Institute for Biologically Inspired Engineering, Harvard University, Boston, MA, USA
* Correspondence: mzw@ucsb.edu

Supplementary Figures

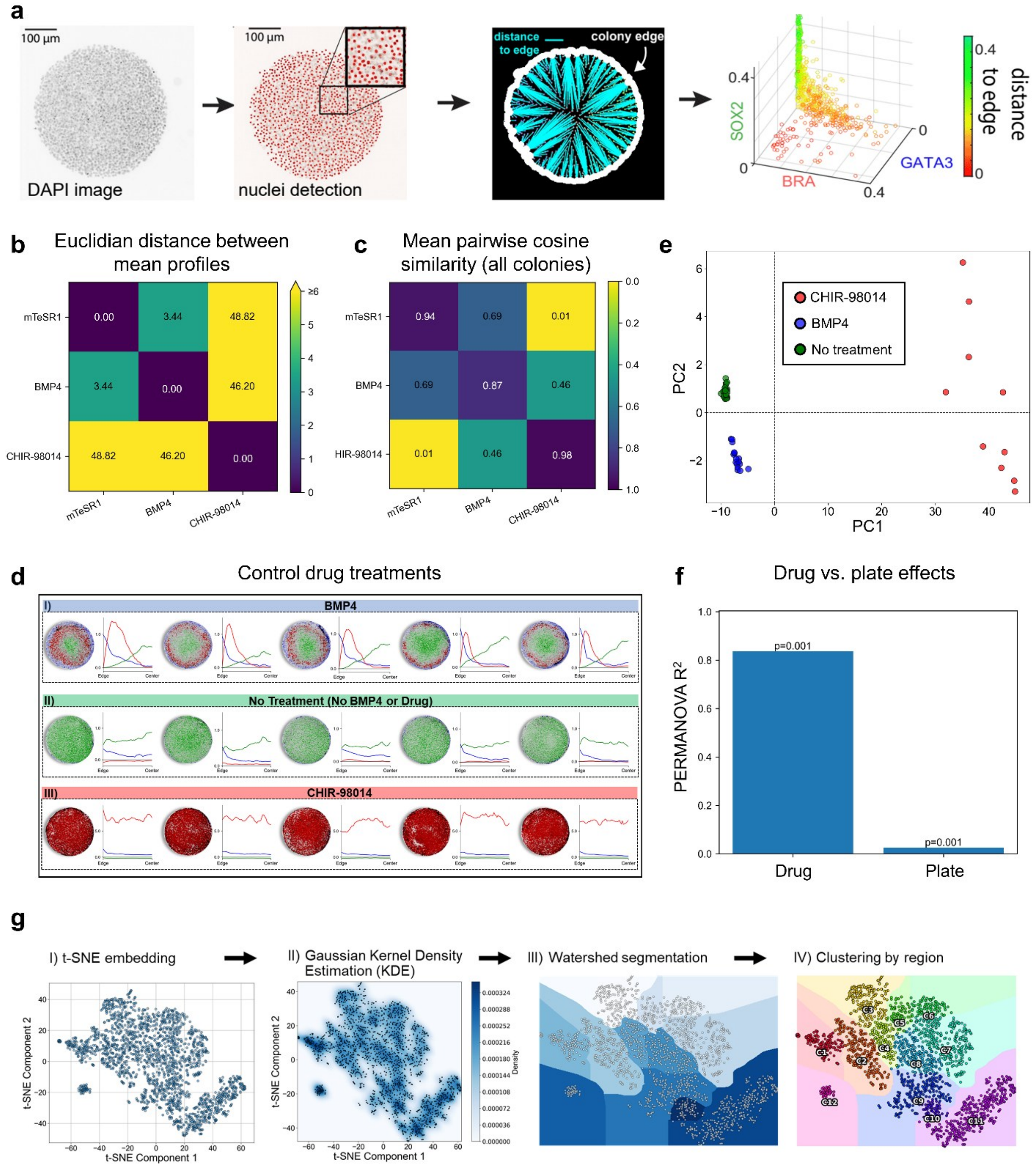

Supplementary Figure 1: Automated image processing and quantification of 2D gastruloid patterning with single-cell resolution. (a) Schematic of the automated image processing pipeline showing single-cell resolution detection. DAPI-stained images of 2D gastruloids are processed to detect nuclei, allowing precise measurement of each cell's location and expression levels within the colony, rather than averaging staining across radial bins. (b) Euclidean distance between mean control drug-treatment profiles. (c) Mean pairwise cosine similarity between all control colonies (higher = more similar; colorbar

inverted). Treatments: mTeSR1, BMP4, CHIR-98014. (d) Images and corresponding vector representations of control treatments. (I) BMP4 treatment leads to canonical radial patterning with GATA3 at the edge, BRA in the intermediate region, and SOX2 concentrated at the center. (II) No treatment (No BMP4 or drug) results in colonies maintaining pluripotency with high SOX2 expression throughout. (III) CHIR-98014 treatment induces BRA overexpression across the colony, disrupting canonical patterning. (e) Principal Component Analysis (PCA) of the 150-dimensional vector data demonstrating the ability to reliably separate control treatments (BMP4, No Treatment, CHIR-98014). (f) PERMANOVA analysis quantifies variance explained by drug ($R^2 = 0.836$, $p = 0.001$) versus plate ($R^2 = 0.025$, $p = 0.001$), confirming that drug effects dominate over batch variability. (g) Schematic of the unsupervised clustering pipeline, which includes: (I) t-SNE embedding of high-dimensional data, (II) Gaussian Kernel Density Estimation (KDE) to smooth the data, (III) watershed segmentation applied to the smoothed density landscape, and (IV) clustering of colonies by region.

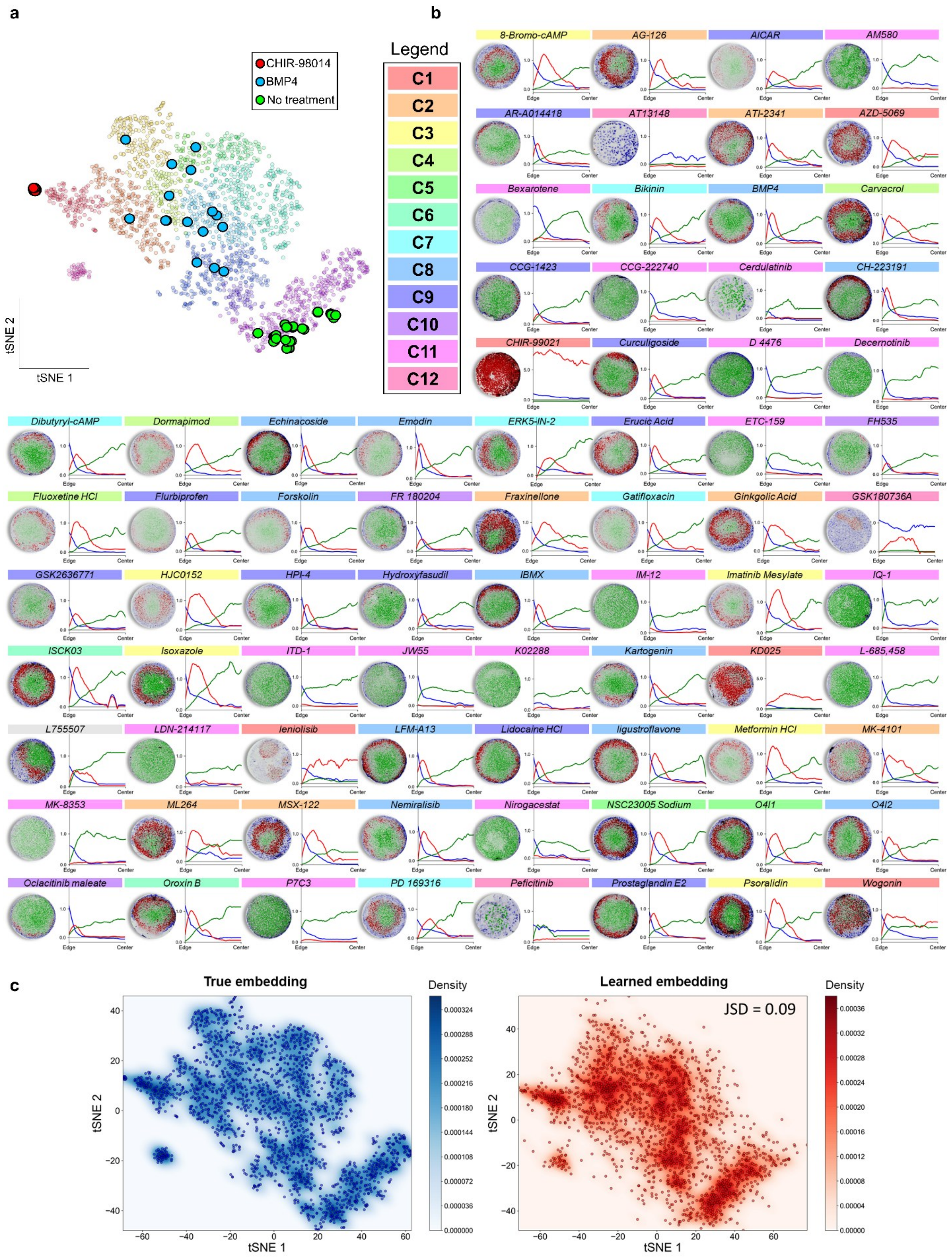

Supplementary Figure 2: Control and drug-induced patterning trajectories in the 2D gastruloid morphospace and validation of embedding accuracy. (a) Location of control treatments (BMP4, CHIR-

98014, No Treatment) within the 2D gastruloid morphospace, showing their distinct clustering patterns that validate the robustness of the clustering method. (b) Representative drug treatments mapped onto morphospace, with each drug's corresponding cluster color-coded at the top. The displayed colonies and their radial intensity plots for GATA3, BRA, and SOX2 highlight the diverse morphological phenotypes induced by different compounds. (c) Density distributions of the true embedding (left, blue) derived from experimental data and the learned embedding (right, red) generated by the neural network. The low Jensen-Shannon Divergence (JSD = 0.09) between the distributions confirms that the learned embedding accurately replicates the experimental morphospace.

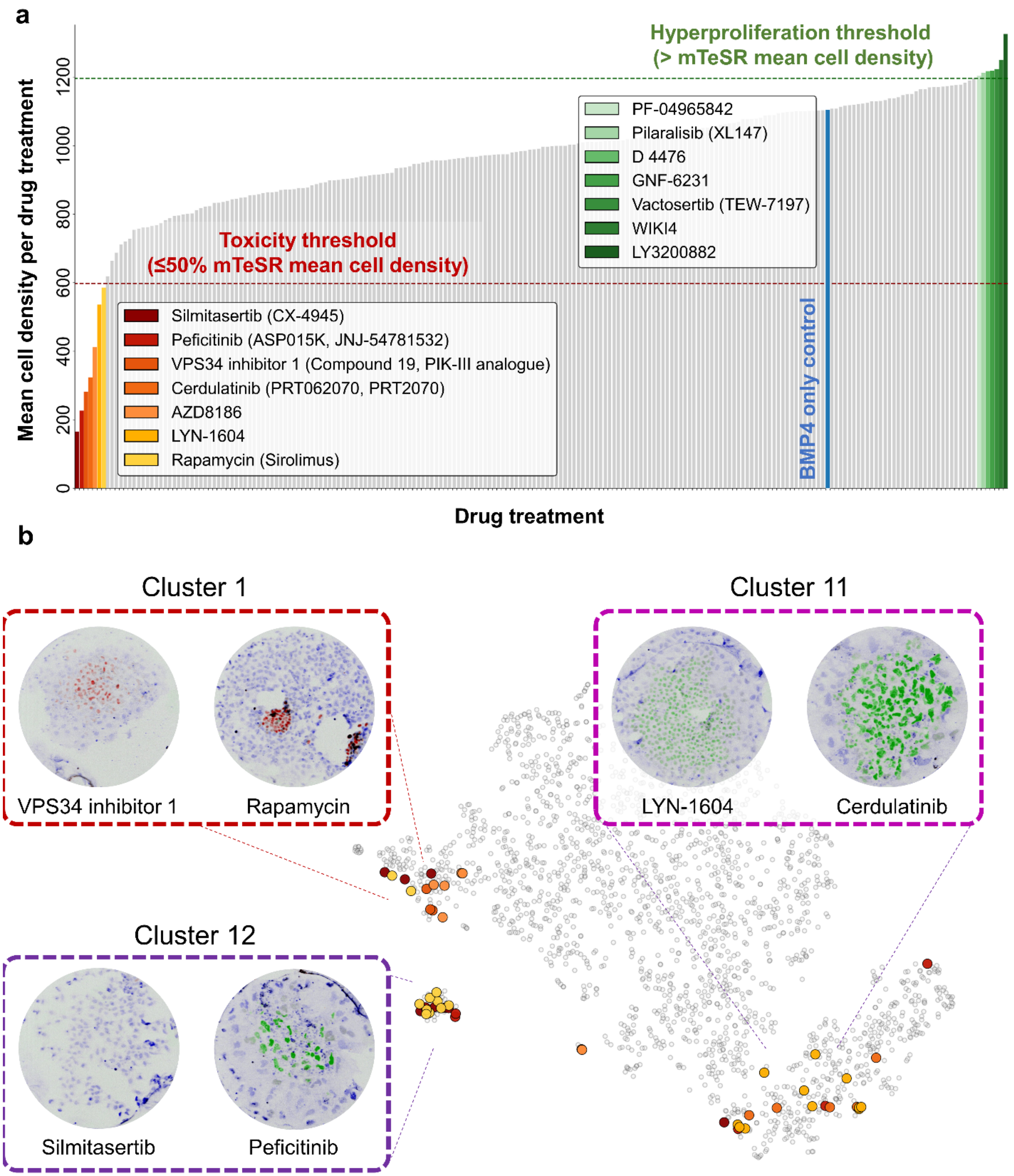

Supplementary Figure 3: Cell density-based toxicity thresholding. (a) Mean nuclei per colony for each BMP4+drug condition. Toxicity threshold (red, ≤50% mTeSR mean) and hyperproliferation threshold (green, >mTeSR mean). (b) Toxic compounds map predominantly to failure modes C1, C11, and C12; insets show representative colonies. The embedding illustrates that overt cytotoxic collapse can be distinguished from density-dependent modulation within the canonical patterning region.

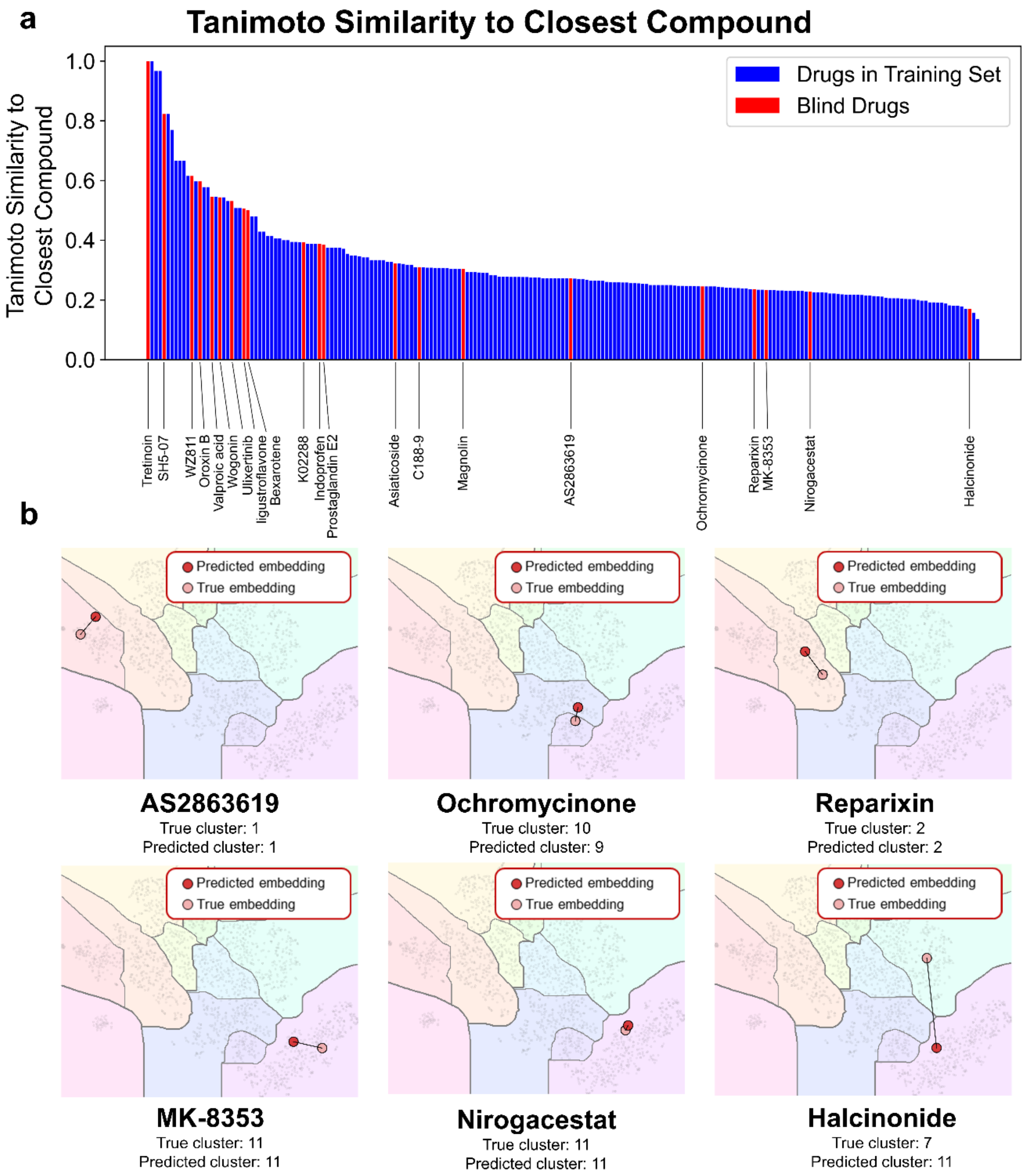

Supplementary Figure 4: FATE-MAP generalizes to structurally diverse compounds. (a) Tanimoto similarity (based on 2048-bit Morgan fingerprints, radius = 2) between each compound and its most structurally similar counterpart in the dataset. Blind test compounds (red), which comprise a random 10% of the total dataset, are distributed across chemical space and include many structurally diverse examples: 12 of the 21 blind compounds have a Tanimoto similarity < 0.5 to any training compound (blue). (b) Predicted and true morphospace embeddings for six blind compounds with the lowest Tanimoto similarity score. Accurate predictions for these structurally diverse compounds support the model's robust generalization to novel chemical structures.

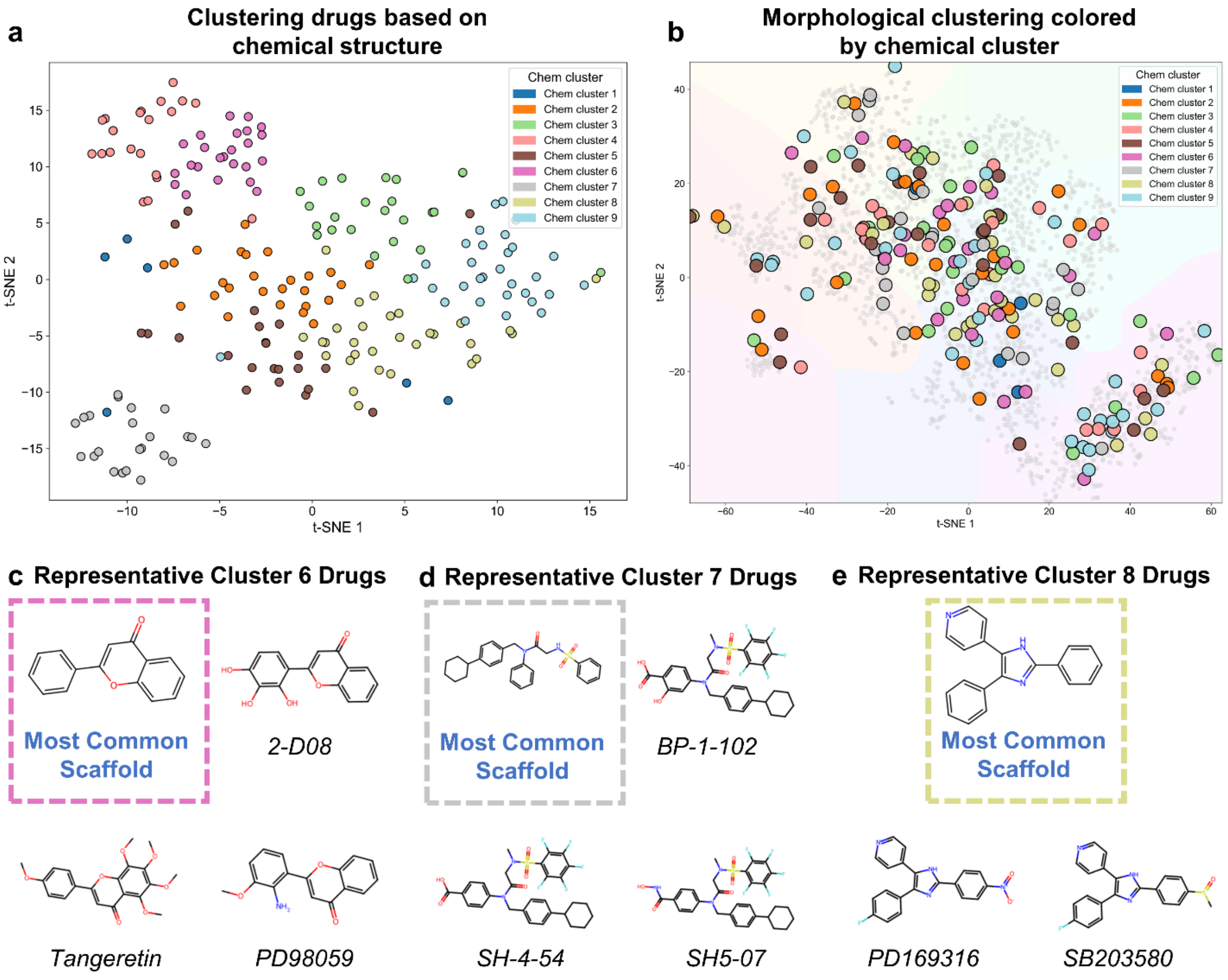

Supplementary Figure 5: Structural similarity alone is poorly predictive of morphological phenotype. (a) t-SNE projection of MACCS fingerprints for all experimentally screened compounds, colored by structure-based chemical clusters identified using KMeans. (b) Morphospace embedding of experimentally profiled compounds, colored by their assigned chemical cluster, overlaid on phenotype-derived morphospace boundaries. Structurally similar compounds are broadly distributed across morphospace, with limited alignment to morphology-defined clusters. (c–e) Representative examples from three chemical clusters (6–8), highlighting the most common Bemis–Murcko scaffold (left) and representative drugs (right). These examples illustrate that scaffold-defined chemical groupings do not reliably correspond to phenotypic similarity, reinforcing the need for neural network–based modeling to uncover structure–function relationships.

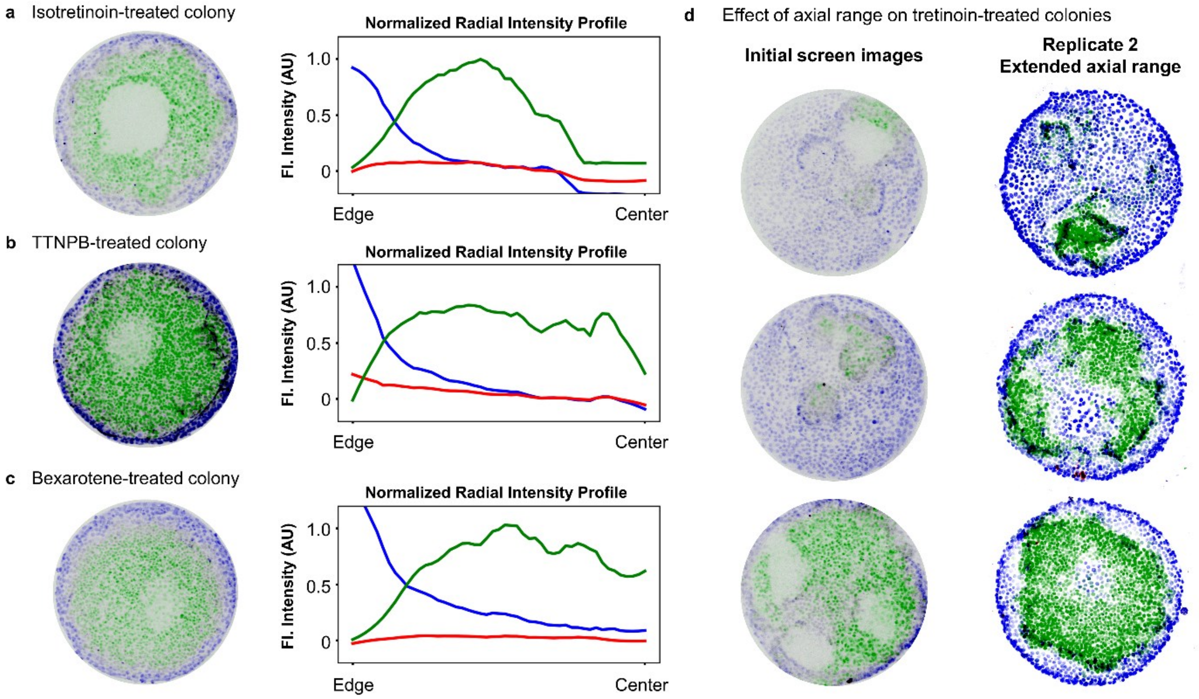

Supplementary Figure 6: Representative retinoid-treated colonies exhibiting vertical protrusions due to increased cell density. Example colonies treated with (a) isotretinoin, (b) TTNPB, and (c) bexarotene show lighter central regions that are not gaps in the cell layer but vertical protrusions arising from confinement within the micropattern. Corresponding normalized radial intensity profiles demonstrate loss of mesodermal identity (BRA, red), consistent with Cluster 11 phenotypes. (d) Tretinoin-treated colonies imaged with an increased focal range (20 μm) show filled centers, validating that the "bald" appearance reflects out-of-plane growth rather than absence of cells.

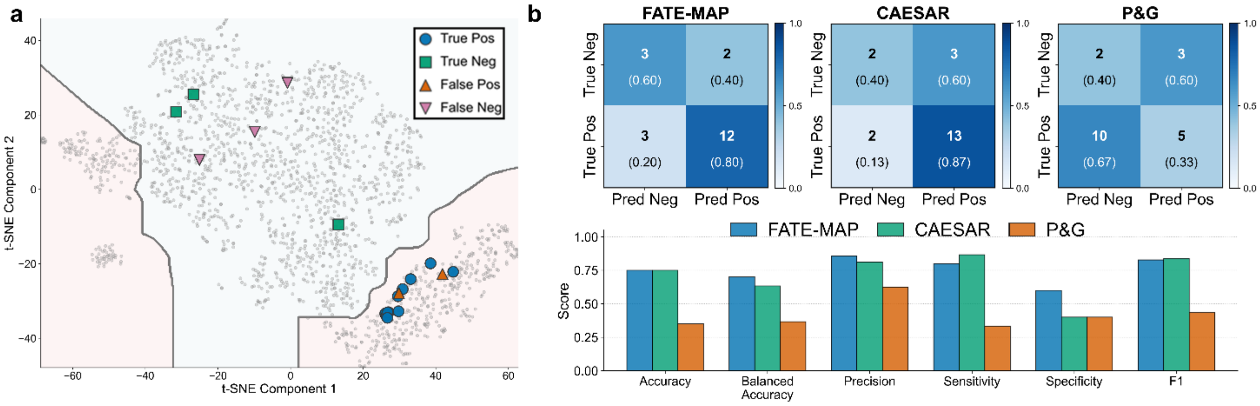

Supplementary Fig. 7: Comparative performance of FATE-MAP and existing *in silico* teratogenicity models. (a) Predicted morphospace embeddings for the 20 compounds held out from training, with points labeled according to whether the model's teratogenicity classification matched the known outcome. Boundaries correspond to the failure-mode (pink) and canonical (blue) morphospace regions. (b) Confusion matrices and performance metrics comparing FATE-MAP to two widely used *in silico* developmental toxicity predictors (CAESAR and P&G) on the same 20-compound benchmark. FATE-MAP matches CAESAR in overall accuracy (75%) and achieves the highest balanced accuracy (85.7%), substantially outperforming the P&G model across all metrics.

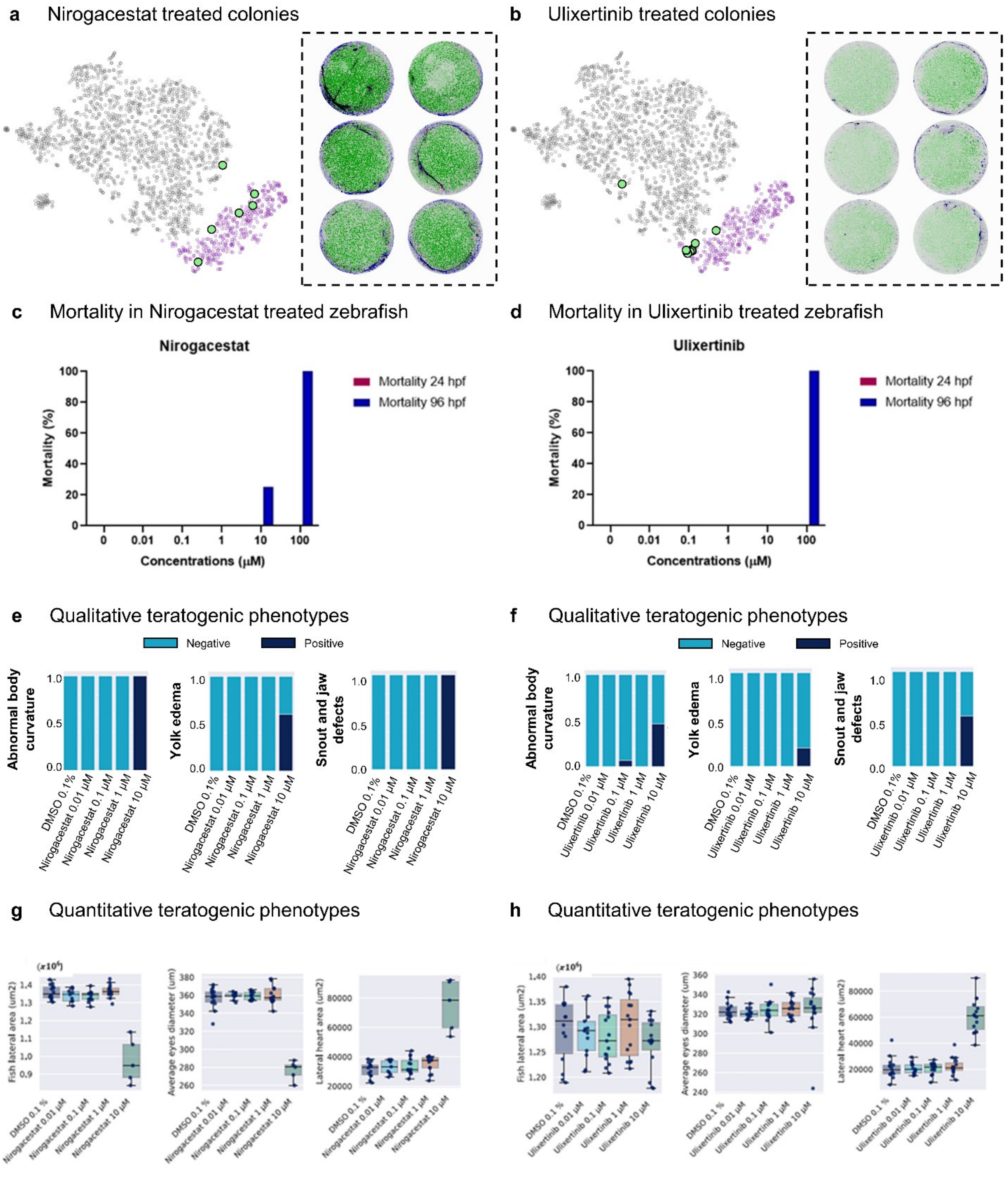

Supplementary Figure 8: Teratogenic assessment of Nirogacestat and Ulixertinib in 2D gastruloids and zebrafish models. (a–b) Nirogacestat (a) and Ulixertinib (b) treated colonies mapped in morphospace (left), both localizing to failure mode cluster C11 (purple). Representative images of treated colonies (right) show disrupted patterning consistent with gastrulation failure. (c–d) Zebrafish embryo mortality following exposure to Nirogacestat (c) and Ulixertinib (d) at varying concentrations, assessed at 24 and 96 hours post-fertilization (hpf). (e–f) Qualitative teratogenic phenotypes observed in zebrafish embryos treated with

Nirogacestat (e) or Ulixertinib (f), including snout and jaw defects, yolk sac edema, and body curvature defects. (g–h) Quantitative phenotyping of Nirogacestat (g) and Ulixertinib (h) treated zebrafish, showing dose-dependent alterations in metrics such as lateral area, lateral heart area, and eye diameter, consistent with developmental toxicity.

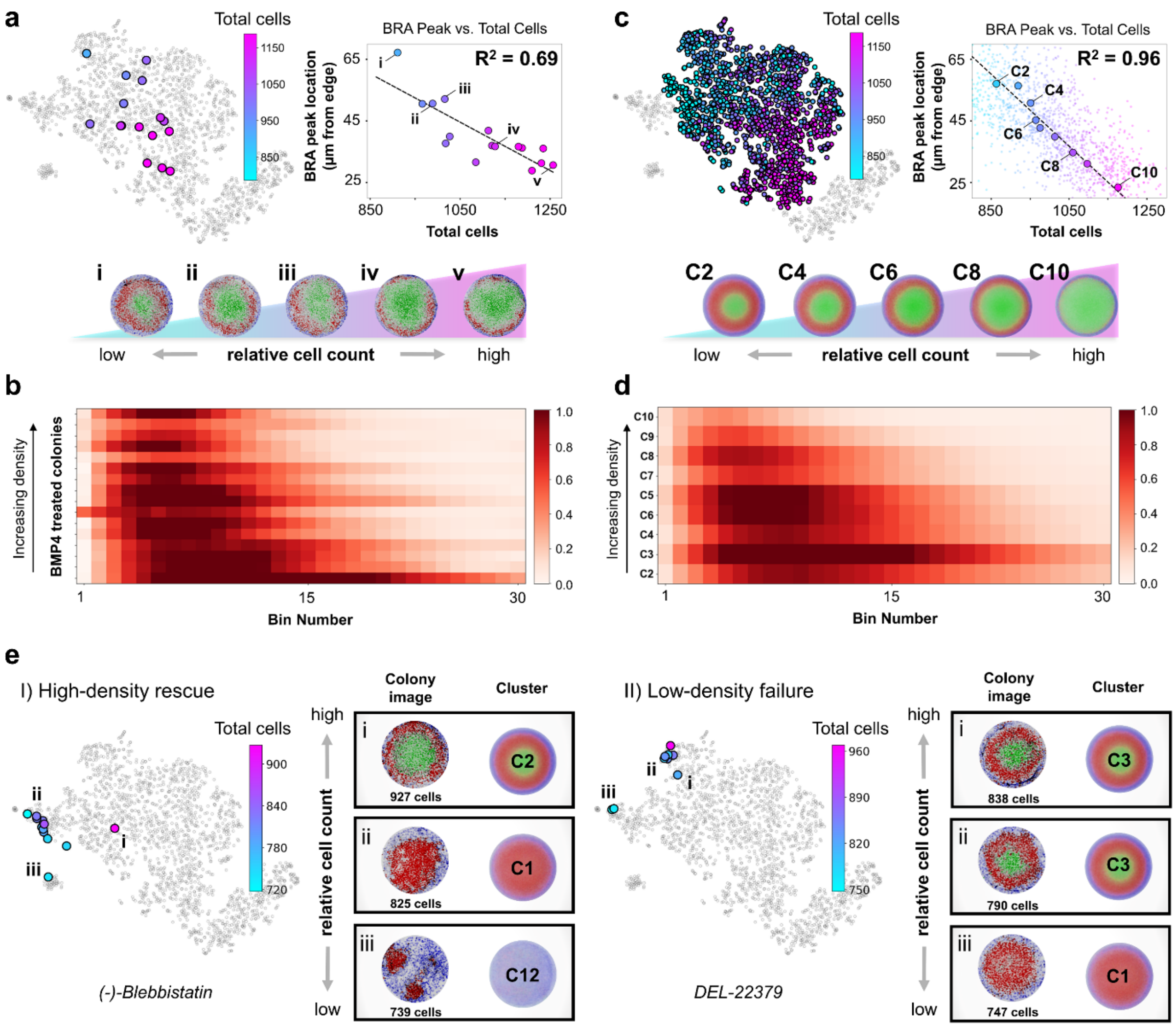

Supplementary Figure 9: Impact of cell density on mesoderm patterning and rescue of drug-induced failure modes in 2D gastruloids. (a) Analysis of cell density effects on patterning in BMP4-treated control colonies. Colonies are plotted in morphospace and colored by cell density, with the inset showing a strong correlation (R^2 = 0.69) between cell density and the inward shift of the BRA (mesoderm) peak. Representative images of highlighted colonies illustrate a clear trend of decreasing mesoderm band thickness with increasing cell density. (b) Heatmap showing edge-to-center mesoderm staining intensity for BMP4-treated controls, ranked by cell density. Results reveal mesoderm expansion and inward shift at lower densities. (c) Colonies from clusters C2–C10 shown in morphospace and colored by cell density. The inset displays the strong correlation (R^2 = 0.96) between BRA peak location and cell density using the mean colony representations for each cluster, with the regression line fit to these mean values. Individual colonies are also plotted and colored by their cell density for comparison. Images of mean clusters further confirm that mesoderm location and width are influenced by cell density across the canonical patterning region. (d) Heatmap of mesoderm width in mean cluster representations of C2-C10, demonstrating that lower cell densities are associated with broader mesoderm bands across the canonical patterning region. (e) The impact of cell density on rescuing or exacerbating gastrulation failure modes. I) High-density conditions can rescue colonies from failure mode clusters (e.g., (-)-Blebbistatin-treated colonies from C1 and C12 are driven back into canonical patterns). II) Conversely, low-density conditions can push drug-treated colonies (e.g., DEL-22379) into failure modes. These results suggest that cell density plays a significant role in modulating patterning outcomes and can potentially correct aberrant gastrulation phenotypes.

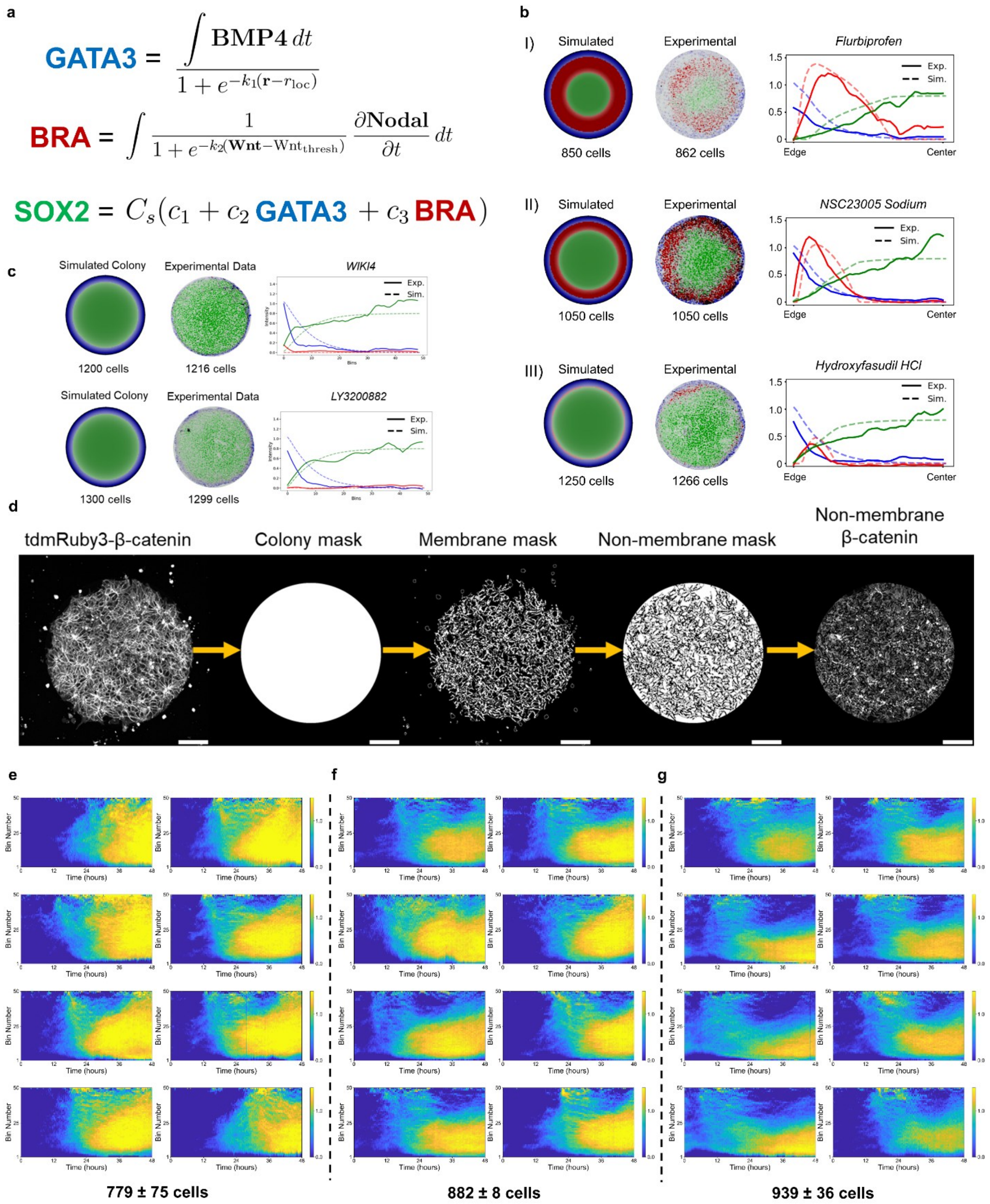

Supplementary Figure 10: Morphogen-based cell fate determination and quantification of Wnt dynamics. (a) Specific morphogen-based cell fate rules used in the simulations, detailing the equations governing the influence of BMP4, Wnt, and Nodal on GATA3, BRA, and SOX2 expression levels, as well as the role of SOX2 stability parameters. For more details, see Star Methods. (b) Additional simulations versus experimental comparisons at various cell densities (850, 1050, and 1250 cells), demonstrating the

model's ability to replicate patterns observed in the screen. Simulated (left) and experimental images (right) with corresponding line plots highlight the spatial distributions of cell fate markers (GATA3, BRA, SOX2). (c) Validation of the model's ability to simulate the effects of small molecule inhibition. Shown is a colony treated with WIKI4, a Tankyrase inhibitor that blocks Wnt/β-catenin signaling. In the simulation, the cell-fate rules remained unchanged, but Wnt autoactivation (s_V) was set to 0, accurately capturing the observed effects of WIKI4 on patterning. Scale bar: 100 μm. (d) Masking strategy for analyzing non-membrane β-catenin, showing the sequential extraction from tdmRuby3-β-catenin images to isolate active signaling components, separating non-membrane β-catenin from the membrane-bound pool. (e-g) Individual kymographs grouped by cell density ranges (low, medium, high) that were used to construct the mean kymographs shown in Figure 4F.

a Potential parameters to modify cell fate

$$\textbf{GATA3} = C_G\left(\frac{\int \mathbf{BMP4}\,dt}{1+e^{-k_1(\mathbf{r}-r_{\mathrm{loc}})}}\right)$$

$$\textbf{BRA} = C_B\left(\int \frac{1}{1+\mathrm{e}^{-k_2(\mathbf{Wnt}-\mathrm{Wnt}_{\mathrm{thresh}})}}\frac{\partial\mathbf{Nodal}}{\partial t}\,dt\right)$$

$$\textbf{SOX2} = C_s(c_1 + c_2\,\textbf{GATA3} + c_3\,\textbf{BRA})$$

b

Parameter	Description
C_G (GATA3 stability)	Scales persistence of GATA3 (effective stability term for GATA3).
C_B (BRA stability)	Scales persistence of BRA (effective stability term for BRA).
C_S (SOX2 stability)	Scales persistence of SOX2 against repression by GATA3 and BRA.
k_1 (GATA3 steepness coeff.)	Sigmoid steepness for GATA3 radial activation.
k_2 (BRA steepness coeff.)	Sigmoid steepness for BRA activation as a function of WNT.
Wnt_{thresh}	WNT level required to trigger BRA induction.
c_1 (Baseline SOX2 level)	Initial/unrepressed SOX2 level.
c_2 (GATA3 repression weight)	Coefficient for GATA3-mediated repression of SOX2.
c_3 (BRA repression weight)	Coefficient for BRA-mediated repression of SOX2.

Supplementary Figure 11: Expanded parameterization of mechanistic cell fate model to evaluate regulatory contributions. (a) Equations defining GATA3, BRA, and SOX2 as functions of all possible stability terms, repression weights, and activation thresholds. (b) Table summarizing model parameters and their biological interpretation.

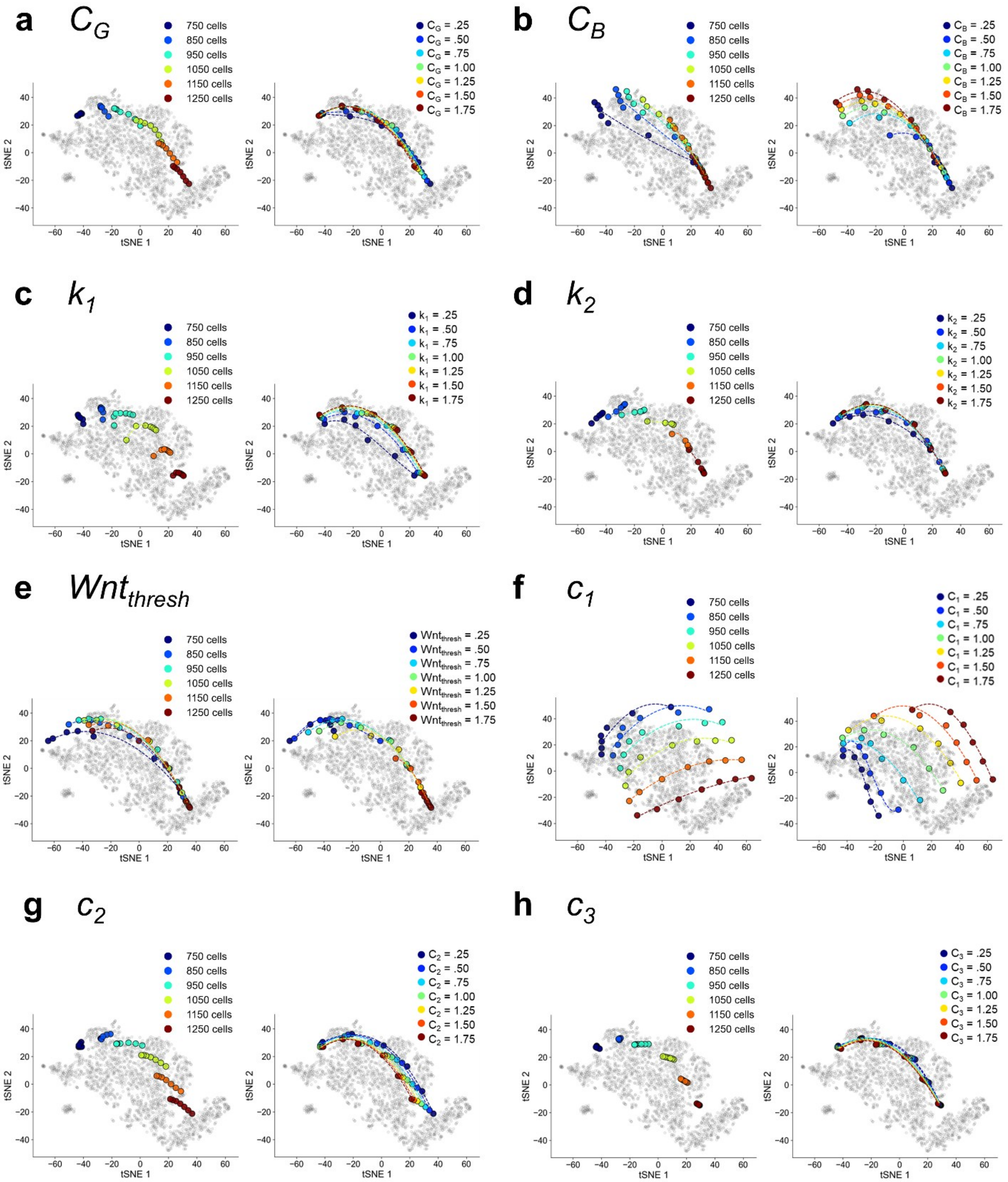

Supplementary Figure 12: Systematic variation of model parameters across morphospace. Simulated colony embeddings projected into morphospace while independently varying each model parameter across a biologically plausible range (0.25–1.75) and across multiple cell densities. Panels show variation of (a) C_G, (b) C_B, (c) k_1, (d) k_2, (e) Wnt_{thresh}, (f) C_1, (g) C_2, and (h) C_3. With the partial exception of C_1, none of the parameters produced trajectories aligned with the major axes of morphospace. C_1 modulates the initial SOX2 level at t = 0 and therefore reflects a trivial shift in initial conditions rather than a regulatory mechanism; because all colonies were seeded from the same initial population, variability in C_1 is unlikely in the experimental screen. This systematic parameter sweep confirms SOX2 stability (C_s) as the principal axis orthogonal to cell density in morphospace.

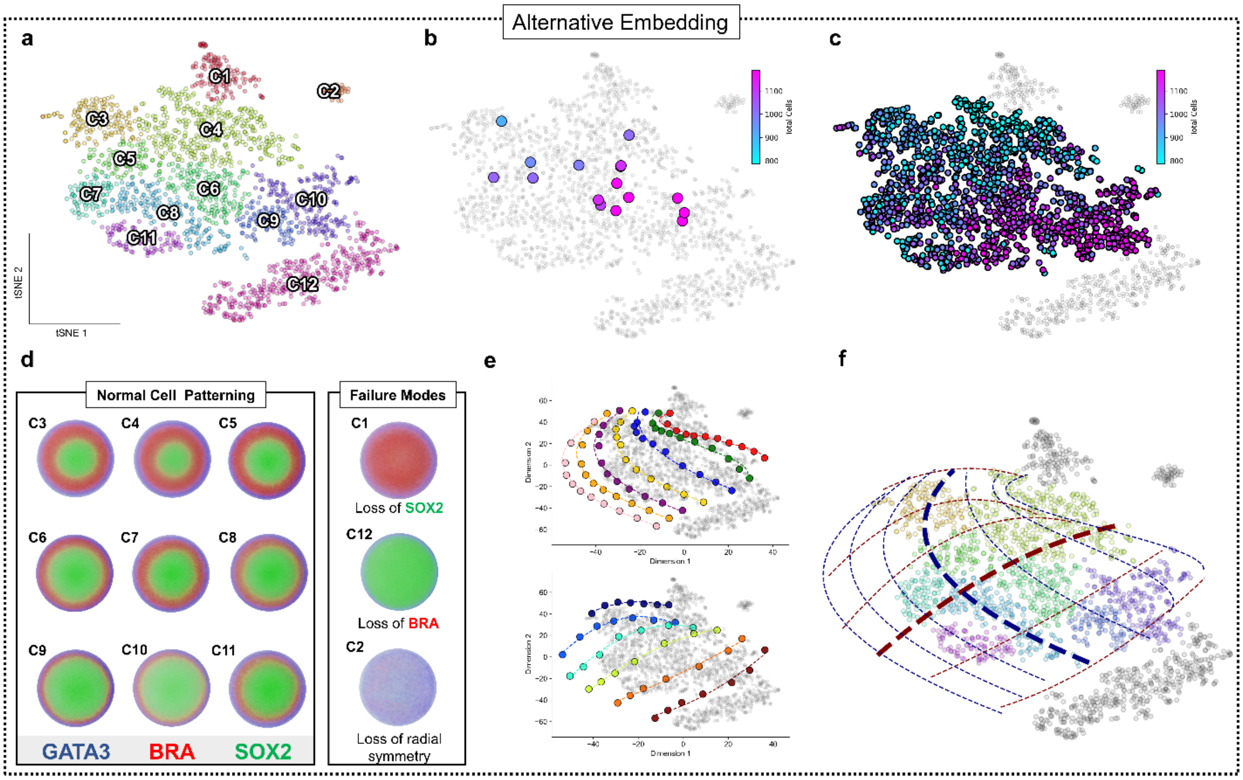

Supplementary Figure 13: Morphospace embedding using different initialization parameters. (a) The alternative morphospace retains the same number of clusters, though their spatial arrangement differs, confirming that clustering results are not specific to a single embedding. (b-c)) Impact of cell density on BMP4-treated controls and canonical patterning clusters (C3-C11) shows the same relationships as the original embedding. (d) Mean cluster images from the alternative embedding consistently capture canonical cell patterning and failure modes (e.g., loss of SOX2, loss of BRA, and loss of radial symmetry). (e) Mapping of simulated colonies onto the alternative morphospace shows that cell density and SOX2 stability still define the two primary axes. (f) The morphospace remains explainable, with fitted polynomials still forming distinct axes, validating that observed trends and explainability are inherent to the data rather than artifacts of specific embedding parameters.

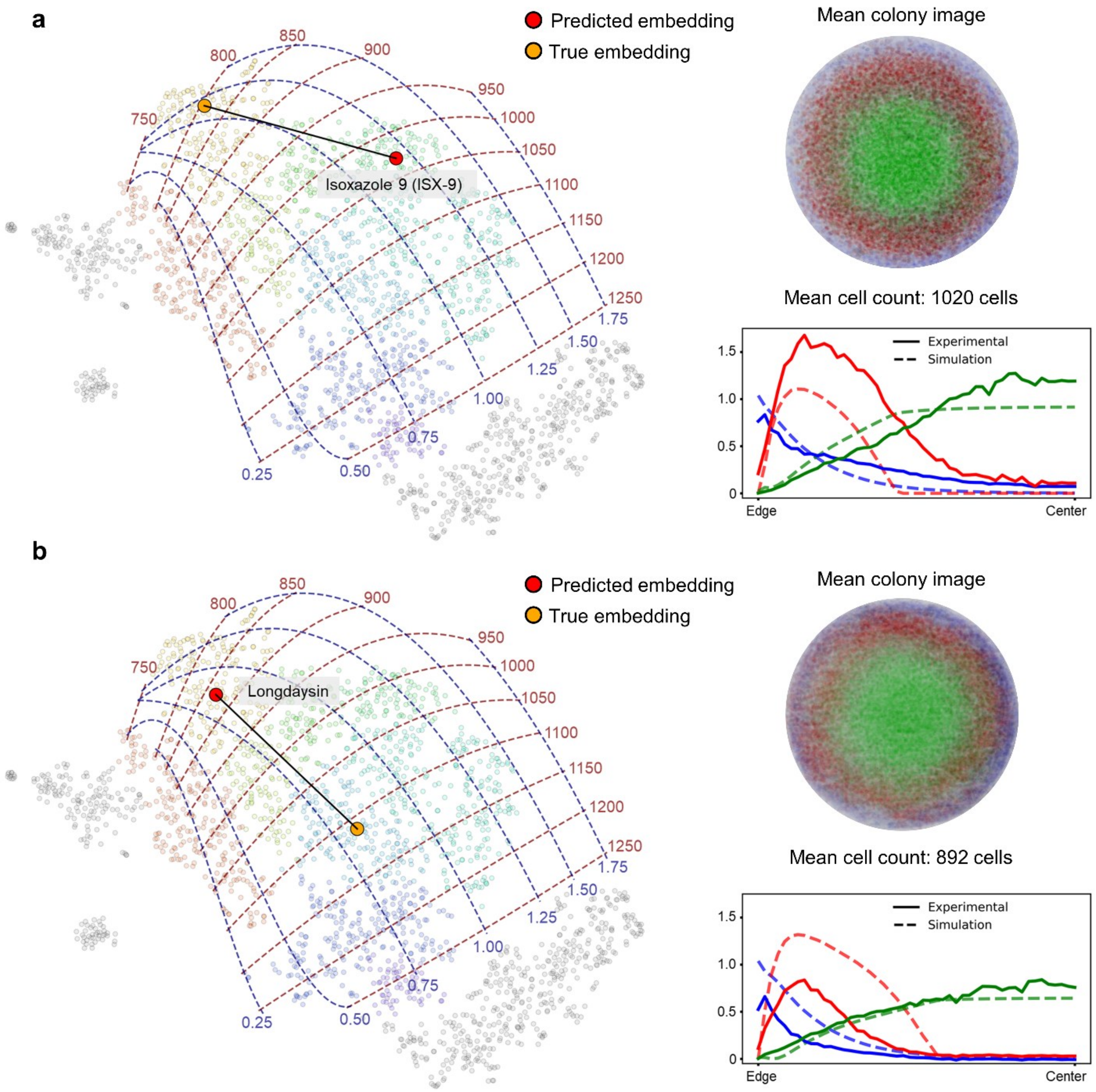

Supplementary Figure 14: Deviations between model predictions and experimental phenotypes reveal mechanistic insights into drug action. (a) Isoxazole 9 (ISX-9)–treated colonies had high cell counts (mean = 1,020), which the morphogen model predicted would yield moderate BRA expression; however, experimental data showed strong BRA induction, consistent with reports that ISX-9 activates Wnt/β-catenin signaling via Axin targeting. (b) Longdaysin–treated colonies had relatively low cell counts (mean = 892), which would predict high BRA levels in simulation, but instead showed reduced BRA experimentally, aligning with evidence that Longdaysin inhibits Wnt/β-catenin signaling.

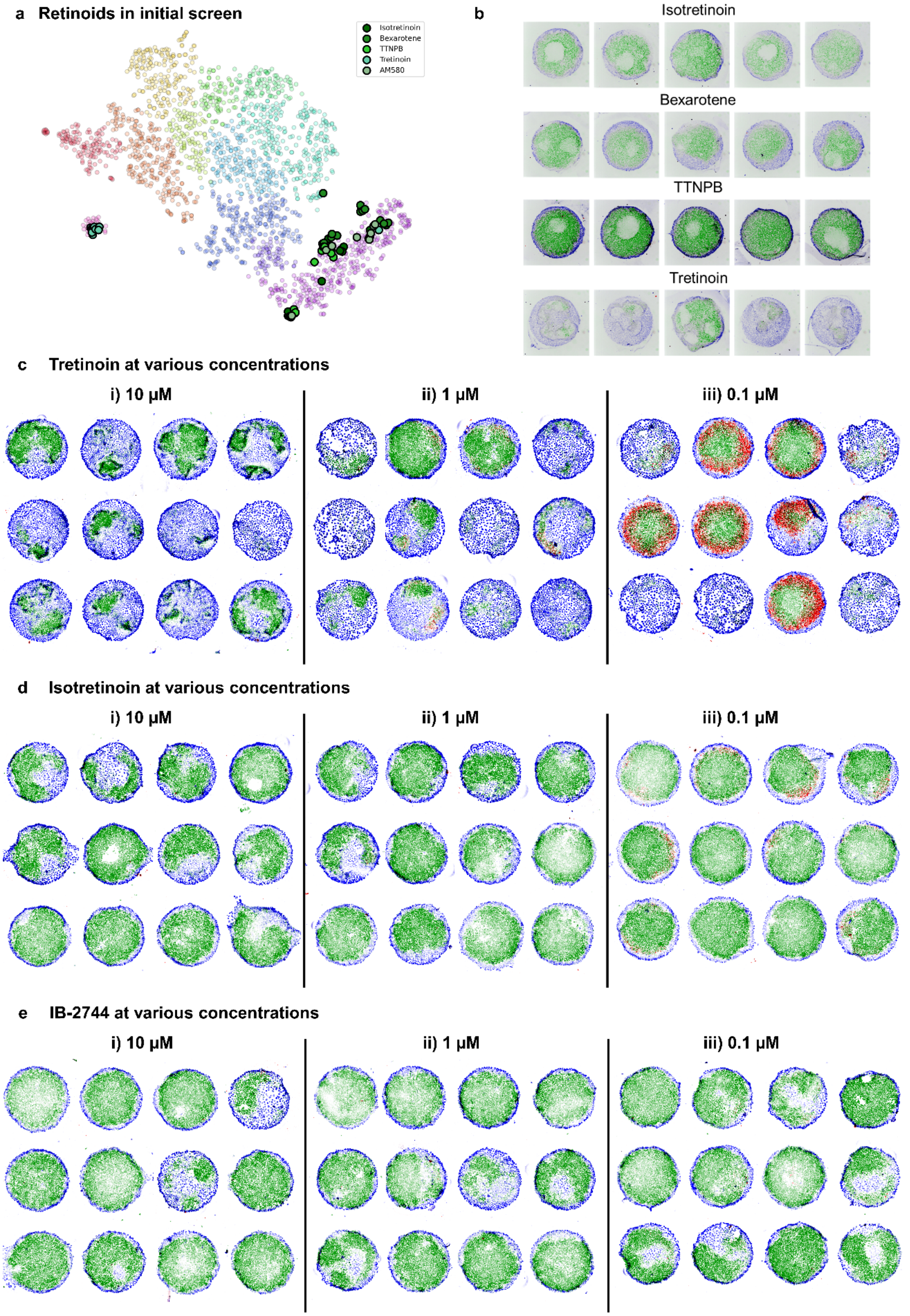

Supplementary Figure 15: Dose–Response Effects of Retinoids on Patterning and Morphology. (a)

Retinoid compounds from the initial screen, including isotretinoin, bexarotene, TTNPB, tretinoin, and AM580, visualized in morphospace. (b) Representative colony phenotypes induced by each compound at 10 µM. (c–d) Follow-up titration experiments for tretinoin and isotretinoin at 10 µM, 1 µM, and 0.1 µM. (e) Titration of an unannotated compound (IB-2744), demonstrating the platform's ability to identify compounds with phenotypes suggestive of shared mechanisms of action.

Supplementary Notes

Supplementary Note 1: Defining thresholds for cluster identification within morphospace

In this study, drug-induced cellular phenotypes are represented in a learned morphospace derived from a two-step modeling framework: (1) a structure-to-phenotype prediction network, which generates a 150-dimensional morphological profile (radial intensity distributions for GATA3, BRA, and SOX2), and (2) a phenotype-to-embedding model that maps this profile into a two-dimensional morphospace for visualization and interpretation.

To interpret predicted drug phenotypes in the context of experimentally observed morphological outcomes, we compare each model-derived phenotype to the mean 150-dimensional experimental phenotype of the corresponding drug treatment (Supplementary Table 6). Similarity is quantified using both root-mean-square error (RMSE) and cosine similarity in the original 150-dimensional feature space, ensuring that model evaluation reflects biologically meaningful morphological structure rather than variation introduced by the embedding. In addition, we visualize predicted phenotypes relative to the mean ± standard deviation of the experimental profiles to assess whether predictions fall within the range of experimentally observed variability.

Once embedded into morphospace, cluster assignments for predicted compounds are determined by directly reusing the watershed-defined boundaries that were originally learned from the full set of experimental colonies. Because the watershed segmentation was applied to the continuous density landscape of the experimental data, it yields spatially contiguous and stable cluster regions. As a result, each predicted phenotype can be assigned to a cluster simply by determining which watershed region it falls into; no new density estimation, re-clustering, or threshold selection is required.

This boundary-reuse capability is a key advantage of the KDE + watershed approach over other commonly used clustering methods (e.g., k-means, DBSCAN, hierarchical clustering), which do not produce continuous, mappable spatial partitions in the embedded morphospace. Methods that operate on discrete point assignments or require cluster number specification are not readily adaptable for in silico classification, whereas watershed segmentation produces a fixed and interpretable partition of morphospace that generalizes naturally to predicted phenotypes.

Thus, the same unsupervised morphological structure discovered from experimental gastruloid data is used consistently for both: 1) defining developmental failure modes, and 2) classifying newly predicted drug phenotypes, enabling scalable *in silico* teratogenicity screening in a manner that is both biologically interpretable and algorithmically well-defined.

Supplementary Tables

Supplementary Table 1. Small molecule screening data

Category	Parameter	Description
Assay	Type of assay	*In vitro* developmental toxicity using micropatterned human embryonic stem cells
	Target	Morphological phenotyping based on presence or absence of GATA3, BRA, and SOX2
	Primary measurement	Radial distribution of cell fate markers (GATA3, BRA, and SOX2) within each colony
	Key reagents	Anti-mouse GATA3 antibody (Thermo Fisher Scientific, Cat #: MA1-028, RRID: AB_2536713), Anti-goat Brachyury antibody (R & D Systems, Cat #: AF2085, RRID: AB_2200235), Anti-rabbit SOX2 antibody (Cell Signaling Technology, Cat #: 3579, RRID: AB_2195767), Alexa Fluor™ 568 Donkey anti-Mouse (Thermo Fisher Scientific, Cat #: A10037, RRID: AB_11180865), Alexa Fluor™ 647 Donkey anti-Goat (Thermo Fisher Scientific, Cat #: A-21447, RRID: AB_2535864), Alexa Fluor™ 488 Donkey anti-Rabbit (Thermo Fisher Scientific, Cat #: A-21206, RRID: AB_2535792), mTeSR™ Plus stabilized feeder-free maintenance medium for human ES cells (STEMCELL™ Technologies, Cat #: 100-0276), CYTOOplate™ 96 RW Custom A (CYTOO Inc., Cat #: A500P650), CellAdhere™ Laminin-521 (STEMCELL™ Technologies, Cat #: 200-0117), Y-27632 (Dihydrochloride), ROCK Inhibitor (STEMCELL™ Technologies, Cat #: 72307), ACCUTASE™ (STEMCELL™ Technologies, Cat #: 07920), Stem Cell Signaling Compound Library (Selleck Chemicals, Cat #: L2100), Human Recombinant BMP-4 (STEMCELL™ Technologies, Cat #: 78211.1)
	Assay protocol	Outlined in "Drug screening protocol" of Methods section
	Additional comments	N/A
Library	Library size	210 compounds with biological activity used for stem cell regulatory and signaling pathway research
	Library composition	
	Source	Stem Cell Signaling Compound Library (Selleck Chemicals, Cat #: L2100)
	Additional comments	Compounds were always added in the presence of 50 ng/mL BMP4 (STEMCELL Technologies, Cat #: 78211.1)
Screen	Format	Micropatterned 96-well plates<br>CYTOOplate™ 96 RW Custom A (CYTOO Inc., Cat #: A500P650)
	Concentration(s) tested	10 µM
	Plate controls	mTeSR™ Plus only (no BMP4 or compound), as well as BMP4 only controls
	Reagent/ compound dispensing system	Micropipette
	Detection instrument and software	Nikon W2 SoRa spinning-disk confocal microscope equipped with incubation chamber maintaining cells at 37 °C and 5% CO2; CellVoyager CQ1 Benchtop High-Content Analysis System (Yokogawa Electric Corporation)
	Assay validation/QC	Standard deviation of radial distribution of cell fate markers (for all controls and compound treatments)
	Correction factors	N/A
	Normalization	Robust scaling (25th–75th percentiles) of fluorescent intensities to mitigate batch effects and outliers
	Additional comments	N/A
Post-HTS analysis	Hit criteria	Absence of BRA was identified as a potentially teratogenic phenotype
	Hit rate	37/210 (17.6%)
	Additional assay(s)	Zebrafish teratogenicity assay
	Confirmation of hit purity and structure	Secondary validation of 3 compounds (1 known teratogen and 2 previously unidentified teratogens) was conducted in a zebrafish teratogenicity assay
	Additional comments	Secondary assay confirmed the teratogenic potential of the 2 previously unidentified teratogens, ulixertinib and nirogacestat
