# Supplementary material for: FATE-MAP predicts teratogenicity and human gastrulation failure modes by integrating deep learning and mechanistic modeling
Source: Nat Commun. 2026 Feb 19;17:3327. doi: 10.1038/s41467-026-69596-6 (PMC13065977; doi:10.1038/s41467-026-69596-6)
Supplement: Supplementary file 3 — Description of Additional Supplementary Files [file 41467_2026_69596_MOESM3_ESM.pdf]

Description of Additional Supplementary Files

File Name: Supplementary Data 1
Description: Validation of FATE-MAP against *in vitro* stem cell methods

File Name: Supplementary Data 2
Description: Validation of FATE-MAP against *in silico* methods

File Name: Supplementary Data 3
Description: Evaluation of model accuracy

File Name: Supplementary Data 4
Description: List of drug centroid locations in the canonical patterning region, ranked by influence on SOX2 stability

File Name: Supplementary Data 5
Description: Guides RNAs for CRISPR tagging β-Catenin

File Name: Supplementary Data 6
Description: Parameter values used in reaction-diffusion model of morphogen spread

File Name: Supplementary Data 7
Description: Parameter values used in morphogen-based cell fate rules

File Name: Supplementary Movie 1
Description: Simulation of density-dependent morphogen dynamics and simultaneous cell fate specification. Simulated spatiotemporal dynamics of Bmp, Wnt, and Nodal over the 48-hour differentiation protocol with corresponding panels for GATA3, BRA, and SOX2 expression at each representative seeding density. These simulations show faster and deeper Wnt/Nodal wave propagation and enhanced BRA penetration as cell density is decreased.

File Name: Supplementary Movie 2
Description: Live imaging of density-dependent Wnt signaling dynamics in human gastruloids. Time-lapse imaging of CRISPR-tagged β-catenin (β-cat) in 2D gastruloids seeded at varying initial cell densities, showing inverse scaling of Wnt wave speed and penetration depth with colony density over 48 hours of differentiation.
