# Supplementary material for: FATE-MAP predicts teratogenicity and human gastrulation failure modes by integrating deep learning and mechanistic modeling
Source: Nat Commun. 2026 Feb 19;17:3327. doi: 10.1038/s41467-026-69596-6 (PMC13065977; doi:10.1038/s41467-026-69596-6)
Supplement: Supplementary file 7 — Reporting Summary [file 41467_2026_69596_MOESM7_ESM.pdf]

nature portfolio

Corresponding author(s): Maxwell Wilson

Last updated by author(s): Jan 27, 2026

Reporting Summary

Nature Portfolio wishes to improve the reproducibility of the work that we publish. This form provides structure for consistency and transparency in reporting. For further information on Nature Portfolio policies, see our Editorial Policies and the Editorial Policy Checklist.

Statistics

For all statistical analyses, confirm that the following items are present in the figure legend, table legend, main text, or Methods section.

n/a	Confirmed	
☐	☒	The exact sample size (*n*) for each experimental group/condition, given as a discrete number and unit of measurement
☐	☒	A statement on whether measurements were taken from distinct samples or whether the same sample was measured repeatedly
☐	☒	The statistical test(s) used AND whether they are one- or two-sided<br>*Only common tests should be described solely by name; describe more complex techniques in the Methods section.*
☐	☒	A description of all covariates tested
☐	☒	A description of any assumptions or corrections, such as tests of normality and adjustment for multiple comparisons
☐	☒	A full description of the statistical parameters including central tendency (e.g. means) or other basic estimates (e.g. regression coefficient) AND variation (e.g. standard deviation) or associated estimates of uncertainty (e.g. confidence intervals)
☐	☒	For null hypothesis testing, the test statistic (e.g. *F*, *t*, *r*) with confidence intervals, effect sizes, degrees of freedom and *P* value noted<br>*Give P values as exact values whenever suitable.*
☒	☐	For Bayesian analysis, information on the choice of priors and Markov chain Monte Carlo settings
☐	☒	For hierarchical and complex designs, identification of the appropriate level for tests and full reporting of outcomes
☐	☒	Estimates of effect sizes (e.g. Cohen's *d*, Pearson's *r*), indicating how they were calculated

Our web collection on statistics for biologists contains articles on many of the points above.

Software and code

Policy information about availability of computer code

Data collection

No custom software was used for collecting the raw experimental data.
Confocal images were acquired using Nikon W2 SoRa spinning-disk confocal microscopy and Yokogawa CQ1 high-content imaging systems under their standard acquisition software (Nikon Elements and CQ1 system software). All raw image files were saved as .tiff formats generated directly by these commercial platforms.
No additional commercial or custom code was required during data collection.

Data analysis	All custom code used for image processing, segmentation, feature extraction, dimensionality reduction, neural-network training, and mechanistic modeling was written in MATLAB (R2021a) and Python (v3.9: NumPy, SciPy, scikit-image, scikit-learn, PyTorch). This includes:<br><br>Custom MATLAB pipelines for nuclear segmentation, mask generation, radial binning, and extraction of 150-dimensional colony phenotype vectors (Methods, pp. 25–26).<br><br>Custom Python scripts for B-catenin reporter processing, Wnt-signaling kymograph generation, and BRA quantification.<br><br>All reaction-diffusion PDE simulations, fate-specification models, neural network models, including:<br>ChemBERTa-based structure-to-phenotype network,<br>parametric t-SNE projection network (y), morphogen reaction–diffusion and fate-decoding simulations.<br>(Methods, pp. 27–36)<br><br>All custom analysis code has been publicly released and is available at the Wilson Lab GitHub repository:<br>https://github.com/mzw-lab

For manuscripts utilizing custom algorithms or software that are central to the research but not yet described in published literature, software must be made available to editors and reviewers. We strongly encourage code deposition in a community repository (e.g. GitHub). See the Nature Portfolio guidelines for submitting code & software for further information.

Data

Policy information about availability of data

All manuscripts must include a data availability statement. This statement should provide the following information, where applicable:

- Accession codes, unique identifiers, or web links for publicly available datasets
- A description of any restrictions on data availability
- For clinical datasets or third party data, please ensure that the statement adheres to our policy

Data Availability

The raw and processed gastruloid images and corresponding morphospace data (radial intensity profiles and cluster assignments) used in all analyses are publicly available at https://max-wilson.mcdb.ucsb.edu/research/gastruloid-morphospace and https://github.com/MZW-Lab/gastruloid_morphospace. These datasets include the binned radial fluorescence intensity profiles for each colony and constitute the complete dataset used to generate the results reported in this study. Additional phenotype categorizations are provided in the Supplementary Information/Source Data file.

Research involving human participants, their data, or biological material

Policy information about studies with human participants or human data. See also policy information about sex, gender (identity/presentation), and sexual orientation and race, ethnicity and racism.

Reporting on sex and gender	This study did not involve human participants, human biological samples, or human participant data. No sex- or gender-related information was collected or applicable.<br>The work used human embryonic stem cell (hESC) lines (H9/WA09) obtained from WiCell, which are not considered human participants and do not contain individually identifiable information.
Reporting on race, ethnicity, or other socially relevant groupings	No human participants or identifiable human data were used. Therefore, no race, ethnicity, or other socially relevant demographic variables were collected or analyzed.
Population characteristics	N/A
Recruitment	N/A
Ethics oversight	Work with human embryonic stem cell lines was conducted in accordance with institutional oversight.<br>As stated in the manuscript (Methods, Cell Culture section), the H9 (WA09) hESC line was obtained from WiCell and maintained under UCSB-approved hESC culture protocols. No research involving human participants or clinical data was performed.

Note that full information on the approval of the study protocol must also be provided in the manuscript.

Field-specific reporting

Please select the one below that is the best fit for your research. If you are not sure, read the appropriate sections before making your selection.

☒ Life sciences ☐ Behavioural & social sciences ☐ Ecological, evolutionary & environmental sciences

For a reference copy of the document with all sections, see nature.com/documents/nr-reporting-summary-flat.pdf

Life sciences study design

All studies must disclose on these points even when the disclosure is negative.

Sample size	For the 210-compound screen, ~10 micropatterned colonies were imaged per drug condition, yielding 2,025 total colonies and ~2 million cells analyzed. This sample size was not predetermined by a statistical calculation; rather, it reflects the throughput capacity of our imaging pipeline and is consistent with established practices for high-content gastruloid screens. The large number of colonies per condition provides sufficient power to identify consistent morphological phenotypes and minimizes variability.<br><br>Sample sizes for zebrafish embryo experiments were not determined using formal a priori statistical power calculations. Instead, sample sizes were chosen based on established practices for zebrafish embryo developmental and teratogenicity screening assays. For each compound and concentration, 16 healthy embryos were treated beginning at 6 hours post-fertilization. This initial sample size allows for reliable detection of reproducible developmental and teratogenic phenotypes while accounting for expected variability in embryo viability and development. Mortality was assessed at 24 and 96 hours post-fertilization, and dead, unhatched, or incorrectly detected larvae were excluded from downstream analysis, resulting in slight variation in the final number of larvae analyzed per condition. The remaining sample sizes were sufficient to quantify both qualitative and quantitative phenotypic outcomes using established scoring and thresholding criteria, and consistent phenotype patterns were observed across embryos within treatment groups.
Data exclusions	No colonies were excluded based on phenotype or experimental outcome.<br>Automated segmentation occasionally removed objects that failed basic nuclear-morphology criteria (e.g., debris or non-cellular fluorescence), but no data points or colonies were excluded post-analysis.<br>The only classification distinction was between true patterning failures and cytotoxic collapse, assessed by total nuclear count, but all colonies were retained in the dataset and mapped in morphospace.
Replication	All experimental conditions were performed in technical replicates across multiple wells and across multiple independent imaging days. High-throughput drug perturbation experiments were conducted across four independent 96-well plates processed on different days, with control conditions (BMP4-only, untreated, and Wnt-activating controls) replicated across plates and in across at least two independent wells within plate. These control conditions exhibited high reproducibility with minimal plate-to-plate variability (Supplementary Fig. 1e–f). Drug-induced phenotypes were consistently observed across replicate colonies within each treatment condition.<br><br>For experiments examining the effect of cell density on Wnt signaling, each density condition was assessed using eight replicate colonies per condition within wells, across three independent wells, with consistent trends observed across replicates.
Randomization	N/A
Blinding	N/A-unsupervised

Reporting for specific materials, systems and methods

We require information from authors about some types of materials, experimental systems and methods used in many studies. Here, indicate whether each material, system or method listed is relevant to your study. If you are not sure if a list item applies to your research, read the appropriate section before selecting a response.

Materials & experimental systems

n/a	Involved in the study	
☐	☒	Antibodies
☐	☒	Eukaryotic cell lines
☒	☐	Palaeontology and archaeology
☐	☒	Animals and other organisms
☒	☐	Clinical data
☒	☐	Dual use research of concern
☒	☐	Plants

Methods

n/a	Involved in the study	
☒	☐	ChIP-seq
☒	☐	Flow cytometry
☒	☐	MRI-based neuroimaging

Antibodies

Antibodies used	Primary antibodies (Dilution ratio: 300:1):<br>GATA3: mouse anti-GATA3 (Thermo Fisher Scientific, Cat. MA1-028, RRID: AB_2536713)<br>Brachyury (BRA): goat anti-Brachyury (R&D Systems, Cat. AF2085, RRID: AB_2200235)<br>SOX2: rabbit anti-SOX2 (Cell Signaling Technology, Cat. 3579, RRID: AB_2195767)<br><br>Secondary antibodies (Dilution ratio: 1000:1):<br>Alexa Fluor 568 Donkey anti-Mouse (Thermo Fisher Scientific, Cat. A10037, RRID: AB_11180865)<br>Alexa Fluor 647 Donkey anti-Goat (Thermo Fisher Scientific, Cat. A21447, RRID: AB_2535864)<br>Alexa Fluor 488 Donkey anti-Rabbit (Thermo Fisher Scientific, Cat. A-21206, RRID: AB_2535792)
Validation	All primary antibodies used in this study are commercially validated for immunofluorescence in human cells by the manufacturers, and each target corresponds to a well-established germ layer or pluripotency marker in human ESCs:

GATA3 is a validated marker of amniotic ectoderm and trophoblast-like fates.

BRA (T) is a canonical and widely validated marker of mesoderm and primitive streak.

SOX2 is a validated pluripotency and epiblast marker.

These antibodies have been extensively used in human 2D gastruloid literature (e.g.,Warmflash et al. 2014; Minn et al. 2020), and the staining patterns observed here match published expression domains for each marker.

No custom antibodies were generated

Eukaryotic cell lines

Policy information about cell lines and Sex and Gender in Research

Cell line source(s)	All experiments were performed using the H9 (WA09) human embryonic stem cell line, obtained from WiCell (Cat. WB06446).<br>A CRISPR-engineered !-catenin–tdmRuby2 reporter derivative of H9 was generated in-house for live imaging analyses
Authentication	H9 lines were purchased directly from WiCell, which performs STR authentication prior to distribution.<br>No additional STR authentication was performed by the authors, as cells were used at early passages and maintained under standard hESC conditions.
Mycoplasma contamination	All hESC cultures used in this study tested negative for mycoplasma contamination using routine screening performed by the UCSB stem cell culture facility. No experiments were conducted with contaminated lines.
Commonly misidentified lines (See ICLAC register)	N/A

Animals and other research organisms

Policy information about studies involving animals; ARRIVE guidelines recommended for reporting animal research, and Sex and Gender in Research

Laboratory animals	Zebrafish (Danio rerio)
Wild animals	*Provide details on animals observed in or captured in the field; report species and age where possible. Describe how animals were caught and transported and what happened to captive animals after the study (if killed, explain why and describe method; if released, say where and when) OR state that the study did not involve wild animals.*
Reporting on sex	Not determined (embryonic stages prior to sexual differentiation)
Field-collected samples	*For laboratory work with field-collected samples, describe all relevant parameters such as housing, maintenance, temperature, photoperiod and end-of-experiment protocol OR state that the study did not involve samples collected from the field.*
Ethics oversight	Experiments were conducted by a commercial provider (ZeClinics) under their institutional animal welfare oversight

Note that full information on the approval of the study protocol must also be provided in the manuscript.

Plants

Seed stocks	*Report on the source of all seed stocks or other plant material used. If applicable, state the seed stock centre and catalogue number. If plant specimens were collected from the field, describe the collection location, date and sampling procedures.*
Novel plant genotypes	*Describe the methods by which all novel plant genotypes were produced. This includes those generated by transgenic approaches, gene editing, chemical/radiation-based mutagenesis and hybridization. For transgenic lines, describe the transformation method, the number of independent lines analyzed and the generation upon which experiments were performed. For gene-edited lines, describe the editor used, the endogenous sequence targeted for editing, the targeting guide RNA sequence (if applicable) and how the editor was applied.*
Authentication	*Describe any authentication procedures for each seed stock used or novel genotype generated. Describe any experiments used to assess the effect of a mutation and, where applicable, how potential secondary effects (e.g. second site T-DNA insertions, mosiacism, off-target gene editing) were examined.*
